# Representation of Hispanic and Latino Orthopaedic Surgery Trainees and Attendings in the United States: A Demographical Analysis

**DOI:** 10.2106/JBJS.OA.25.00349

**Published:** 2026-08-18

**Authors:** Emmanuel Brito, Nathan Sherman, Guy Paiement

**Affiliations:** 1 Department of Orthopaedic Surgery, University of Arizona, Tucson, Arizona; 2 Department of Orthopaedic Surgery, Tucson Orthopaedic Institute, Tucson, Arizona; 3 Department of Orthopaedic Surgery, Cedars-Sinai Medical Center, Los Angeles, California

## Abstract

**Introduction:**

In 2021, the US Hispanic and Latino (H&L) population made up 19% of the national population, an increase from 14% in 2006. There is limited literature assessing the trends of H&L representation among residents, fellows, and attending physicians in orthopaedic surgery. We sought to analyze their current representation in light of recent changes in national demographics.

**Methods:**

Demographic data for the US population was obtained from the decennial Censuses. Race and ethnicity data for in-training residents from 2006 to 2021 were extracted from the Graduate Medical Education Census from the American Medical Association. Similar data were obtained from the 2008 and 2018 AAOS (American Academy of Orthopedic Surgeons) Censuses of fully trained orthopaedic surgeons. Representation was analyzed for the following groups: H&L, Black, Asian, Native American, Multiracial, and White. H&L fellows representation per subspecialty (adult reconstruction, foot and ankle, hand, oncology, sports medicine, spine, trauma, and pediatrics) was also analyzed.

**Results:**

A total of 57,187 orthopaedic surgery residents were in training over the 15-year study period (2006-2021), 2,812 (4.9%) identified as H&L. Total number of orthopaedic residents in-training grew by 36% from 2006 to 2021 (3,305 to 4,616). H&L residents doubled from 118 to 281 to comprise 6.1% of trainees. Representation for H&L trained orthopaedic surgeons grew from 484 (1.9%) in 2008 to 663 (2.2%) in 2018, a 37% increase in absolute number and 16% as percentage of the orthopaedic workforce.

**Conclusion:**

Across the 15-year study period, H&L representation in orthopaedic surgery moderately increased at all levels but remained low relative to the H&L population. H&L resident representation relative to H&L national demographic weight is significantly low (p < 0.001) compared with other ethnic/racial groups. H&L residents were represented at a rate 3 times lower than White residents and almost 7 times lower than Asian residents when compared with the H&L national population.

## Introduction

From 2006 to 2021, the US Hispanic and Latino (H&L) population increased by 37%, now making up 19% of the nation^1^. US regions with historically low H&L presence have also seen a rise of their H&L populations^2^. By contrast, the percentage of H&L trained orthopaedic surgeons has only increased from 1.9% to 2.2% of the national orthopaedic workforce from 2008 to 2021^3^.

Orthopaedic surgery remains one of the least diverse medical specialties and consistently has the lowest proportion of H&L residents^3-9^. This discrepancy may affect patient outcomes in select populations, as ethnic/racial concordant health care has been demonstrated to result in more efficient communication, higher patient satisfaction, and improved outcomes^10-12^. Orthopaedic care parallels other fields of medicine in that minority patients are less likely to undergo timely surgical treatment for indicated procedures, like a total knee arthroplasty, and have increased postoperative complications with increased readmission rates^13-22^.

Although representation in healthcare is multifaceted, there remains a discordance in H&L representation in orthopaedic surgery relative to the H&L national population. While the awareness of demographic disparities exists within orthopaedic surgery, there is currently no published literature directly analyzing the longitudinal representation of H&Ls in our field. We aim to establish the first multitier baseline and to assess the state of H&L representation in orthopaedic surgery at the resident, fellow, and attending levels relative to the growth of the H&L population. We hypothesize that there remains a lower H&L representation in orthopaedic surgery relative to the H&L weight in the US population.

## Methods

After Institutional Review Board exemption, demographic data for the US population were obtained from the US Census Bureau^23^. Race and ethnicity data for in-training residents and fellows were extracted from the Graduate Medical Education (GME) Census published in the *Journal of the American Medical Association* from 2006 to 2021. The GME Census defines race and ethnicity in concordance with federal standards of the Office of Management and Budget. They define race/ethnicity together as a self-identification with one or more social groups based on appearance and ancestry. These categories include H&L, Black, Asian, Native American, Multiracial, and White^24^. For the purpose of this study, the Multiracial and Native American groups were excluded from the primary comparative analysis due to their limited sample sizes within the AAOS data and data collection timeline. Specifically, the ‘Multiracial’ designation was only introduced as a distinct category in the GME Census in 2014. In addition, the national registry data sets used do not include a granular breakdown of their ethnic composition (i.e. Black/White, Asian/White, or Black/Hispanic). Excluding this category would ensure data homogeneity and reduce speculative classification which could result in a relative underestimation across select demographic groups.

Resident data were collected sequentially from 2006 to 2021 through the National Resident Matching Program annual GME survey of newly matched trainees in Accreditation Council for Graduate Medical Education (ACGME)-accredited programs. This database reportedly adds approximately 19,000 new residents to its database per application year. Resident ethnic/racial groups data from 2010 to 2020 were matched and compared with the US Censuses of 2010 and 2020.

χ^2^ analysis was then used to assess whether the distribution of residents across all ethnic/racial groups differed significantly between the 2 time points. A resident to population ratio standardized per 100,000 people of their own ethnic/racial group was calculated to determine whether H&L residents were underrepresented compared with their Asian and White colleagues. A χ^2^ analysis was also performed to evaluate the observed distribution of surgeons based on population size and change. Representation for each group (H&L, Black, Asian, and White) was calculated as percentages of the total orthopaedic surgery residents in each academic year. H&L fellows by subspecialty (adult reconstructive, foot and ankle, hand, oncology, sports medicine, spine, trauma, and pediatrics) were compared over the 15 years between 2006 and 2021. Attending orthopaedic surgeon demographics were obtained from the 2008 and 2018 AAOS Censuses^25^. A ratio of orthopaedic attendings for each race was calculated per 100,000 people of their respective ethnic/racial group from 2008 to 2018.

### Statistical Analysis

Statistical analysis was performed using SPSS version 28 (IBM Corporation, 2021, Armonk, NY). Descriptive and univariate analysis was performed using the Pearson χ^2^ or Students *T* test. Statistical significance was defined by p < 0.05. All statistical calculations were reviewed by a trained statistician.

## Results

### Residency

**Fig. 1:**
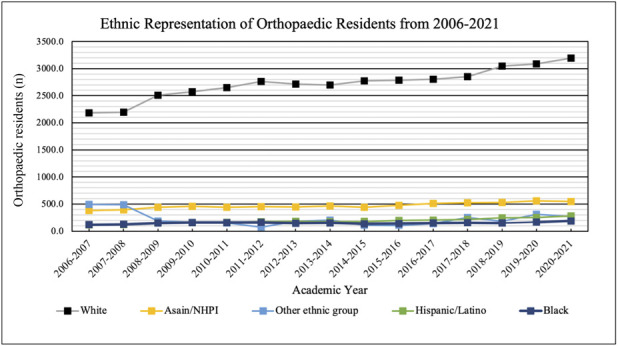
Resident ethnic demographics from 2006 to 2021.

**Fig. 2:**
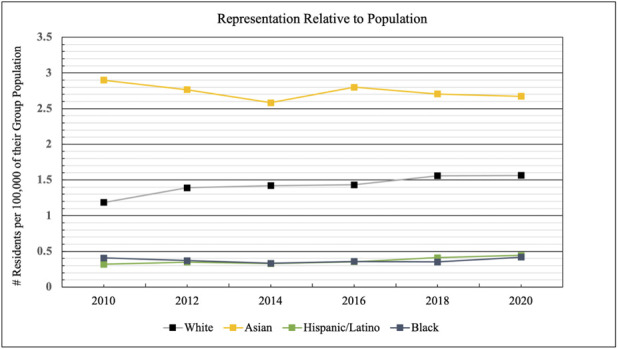
Resident representation relative to their ethnic weight in the population from 2010 to 2020.

A total of 57,187 orthopaedic residents were identified between 2006 and 2021. The number of orthopaedic residents in training per year grew from 3,305 in 2006 to 4,616 in 2021, a 36% increase. The number of H&L orthopaedic residents grew from 118 (3.6%) in 2006 to 281 residents (6.1%) in 2021, an increase of 138%. The number of Black residents grew from 121 (3.7%) to 189 (4.1%), while White and Asian residents grew from 2,185 (66.1%) to 3,196 (69.2%) and from 380 (11.5%) to 549 (11.9%), respectively (Fig. 1 and Supplemental 1). H&L representation in residency relative to their weight in the national population was low from 2010 to 2020, as it increased from 0.32 to 0.44 residents per 100,000 H&L people. This is significantly lower compared with White (1.2 to 1.6 per 100,000 people) and Asian (2.9 to 2.7 per 100,000 people) representation relative to their respective ethnic/racial group (p =< 0.001) (Fig. 2).

### Fellowship

Analysis of orthopaedic residents pursuing a fellowship revealed that H&L represented 5.1% of all matriculants from 2006 to 2021. Black fellows during this period comprised 231 individuals (4%), White was 4,245 individuals (74.1%), and Asian 958 individuals (16.7%), respectively. H&L representation per year more than doubled from 10 fellows (3.8%) in 2006 to 28 fellows (7.7%) in 2021. (Table I).

**TABLE I:** Ethnic/Racial Representation by Fellowship from 2006 to 2021

	Total # of Fellows	Hispanic and Latino	Black	Asian	White
Adult reconstruction	538	6%	3%	21%	58%
Foot and Ankle	210	6%	4%	16%	65%
Hand	1,906	4%	3%	16%	69%
Oncology	188	9%	5%	20%	53%
Sports medicine	2,416	4%	4%	11%	71%
Spine	437	6%	7%	24%	49%
Trauma	205	2%	3%	13%	70%
Pediatrics	476	6%	5%	15%	63%
Total	5,728	5%	4%	17%	74%

Multiracial and Native American groups were excluded for simplification of results. Total percent does not add up to 100%.

### Attending Orthopaedic Surgeons

**Fig. 3:**
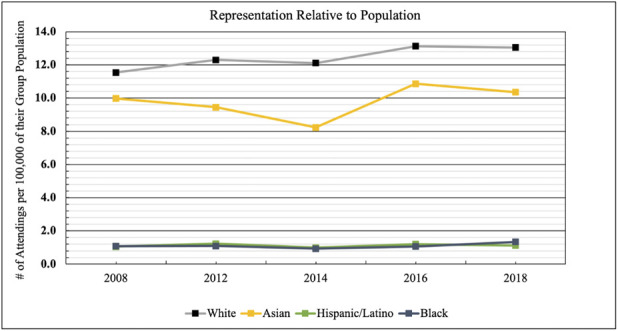
Number of attendings of a given ethnic group per 100,000 of their respective group from 2008 to 2018.

The total number of orthopaedic attendings grew from 25,464 in 2008 to 30,127 in 2018 (18.3% increase). The number of H&L attendings increased from 484 (1.9%) to 663 (2.2%) (37% increase) during the same period according to the AAOS Censuses. The number of attendings per 100,000 people of the same ethnic/racial group were calculated, and H&L remained at the same ratio (1.1 per 100,000) from 2008 to 2018 while all other groups increased their numbers (Supplemental 2). Black attending representation per 100,000 people increased from 1.1 to 1.3, Asian from 10.0 to 10.4, and White attendings from 11.4 to 13.1 of their respective race/ethnicity (Fig. 3).

## Discussion

The aim of this study was to assess the change in H&L representation in orthopaedic surgery relative to national demographic trends from 2006 to 2021. While H&L representation has more than doubled in absolute numbers, their representation remains markedly low relative to their national demographic weight when compared with other ethnic/racial groups at each level of training. Statistical analysis confirms H&L underrepresentation despite these recent increases (Fig. 2). Similar findings have been demonstrated across both medical and surgical residencies^26^.

Fellowship training has the largest incongruity in representation. H&L trainees only make up 5.1% of matriculated fellows, and their representation varied greatly by subspecialty. There is also a stark underrepresentation of minorities in fellowship training relative to their weight in the general population, consistent with trends observed in residency. Analyzing representation at the fellowship level is critical, as this is the primary educational bottleneck for H&L trainees based on our findings. Subspecialty training has become ubiquitous across residencies^31,39^ and can dictate career trajectories, academic leadership opportunities, and involvement in subspecialty societies. As such, fellowship selection data can help identify deficits in subspecialty mentorship or exposure during residency.

At the attending level, there was an increase in representation from 1.9% to 2.2% of the total workforce. When standardized per 100,000 individuals of their respective ethnicity/race, H&L surgeon representation remained at 1.1 per 100,000 H&L people, and this ratio did not change from 2008 to 2018. Attending level representation per 100,000 people of the same race is approximately 3 times lower than for White surgeons and 7 times lower than for Asian surgeons. (Fig. 3) This representation trend is also seen in both residency and fellowship.

The decision to pursue a career in orthopaedic surgery is multifactorial. A possible explanation of the underrepresentation of H&L could be the younger median age of the H&L population. H&Ls have the youngest median age at 30 years per the National Census. This is to be contrasted with the national and White median age of 38.9 and 40 years, respectively. Asian median age is 34.7 and Black 35.1 years. This difference could partially explain the representation gap as fewer H&L individuals have reached the age for medical school, residency, and attendingship. This would also indirectly affect the mentorship of younger generations for such a career. Socioeconomic and familial responsibilities can additionally influence their decision to go to medical school or choose a relatively long residency training^27^. This trend is likely further increased because of immigration and higher natality rates^28,29^.

Although this is a complex issue that has been attributed to lack of exposure, lack of mentorship, and challenging socioeconomic conditions^28,29^, underrepresented minorities tend to be directed toward primary care specialties^30-32^. Critical facilitators in increasing representation are pipeline programs such as Nth Dimensions and the Perry initiative which connect medical students through funded summer research and direct mentorship from national orthopaedic leaders. Yielding a 93% residency match rate among their scholars warrants a discussion about expanding pipeline pathways such as these to increase minority attrition^28,34^.

While this challenge has been established in our field, providing an updated objective measure is necessary in assessing our progress. The literature evaluating barriers to recruiting diverse trainees indicates a cyclical recruitment deficit in that applicants gravitate toward institutions with established diversity and culturally concordant mentorship at the attending level. In addition, an overlooked rising issue is the cost of audition subinternship rotations. Away rotations have evolved into a mandatory variable for match success, with over 54% of matched applicants securing a position at their home institution or at a program they completed an away rotation. The average orthopaedic applicant will easily spend $3,000 to $4,000 to cover housing, travel, application fees, etc. This hurdle for socioeconomically disadvantaged and underrepresented minorities who lack an affluent safety net or institutional stipends significantly narrows their match probability^35-37^.

This issue directly affects patient care as prior studies have demonstrated that race-concordant physician-patient interactions result in higher patient satisfaction and improved communication^13,14,16,33,34^, warranting the continued attention of national, regional, and state organizations^16,30-43^.

### Limitations

This study has limitations as a retrospective review of a database. There is a lack of data on retention rate at the resident and fellow level of training. Although we were able to collect the number of matched trainees, there can be variability in residents that complete training which may not be accounted for. We were also not able to determine if a residency applicant was a US versus international medical graduate, osteopathic or allopathic graduate, or had a home orthopaedic program which could affect match rates. There were also limitations on data acquisition for the national population in each racial group as reported by the US Census Bureau. There are inherent limitations and mathematical challenges with Census data, and collection and extrapolations methods may have changed over the course of our study period. One such change occurred in 2014 where the category of “multiracial” became a separate demographic group, which may affect racial category subjective reporting before and after this change. Previous database iterations as used in this study from the ACGME and the AAOS frequently comingle the distinct categories of race and ethnicity into a single survey item. This structural limitation creates inherent risk of misclassification or under-reporting among individuals who identify as ethnically H&L but racially White or Black. Another limitation was the nature of the collection period for the decennial national Census and the AAOS Census. The earliest publicly available Census for the AAOS was 2008 while the latest being 2018, which is also limited by the varying number of respondents to the online survey. These surveys for the ACGME, AAOS, and decennial Census are additionally subjective in nature and limited due to self-reported information, such as ethnicity and race, which may be variably reported.

## Conclusion

Across the 15-year study period, H&L representation in orthopaedic surgery has moderately increased at the resident and fellowship level but remained stagnant at the attending level. Ethnic/race representation among residents relative to their respective national demographics reveals large significant differences between groups (p =< 0.001). When standardized, H&L residents were represented at rates less than one-third of White residents and almost 7 times lower than Asian residents. There is no consensus on how closely ethnic/racial representation in orthopaedic surgery should match the ethnic/race composition in the general population.

## Appendix

Supporting material provided by the authors is posted with the online version of this article as a data supplement at jbjs.org (https://links.lww.com/JBJSOA/B299). This content was not copy-edited or verified by JBJS.
